# Evaluation of real-world follow-up methods and their association with adherence and safety in adjuvant breast cancer therapy: a retrospective study

**DOI:** 10.3389/fonc.2025.1661727

**Published:** 2025-09-30

**Authors:** Na Li, Qian Zhang, Xiaomei Yang, Xiaoli Li, Xixi Tian, Wei Li, Lili Ren, Hua Yang

**Affiliations:** 1 Department of Medical Oncology, Affiliated Hospital of Hebei University, Baoding, Hebei, China; 2 Hebei Key Laboratory of Cancer Radiotherapy and Chemotherapy, Baoding, Hebei, China; 3 Teaching Office, Affiliated Hospital of Hebei University, Baoding, Hebei, China

**Keywords:** breast cancer, treatment compliance, digital follow-up, self-management, patient-reported outcomes

## Abstract

**Aim:**

To compare the associations between different follow-up management methods—telephone, WeChat, and mini-program—and treatment compliance, safety, quality of life, and self-management in breast cancer patients receiving adjuvant therapy.

**Methods:**

This retrospective study reviewed clinical and follow-up records of patients with newly diagnosed breast cancer who underwent adjuvant therapy after radical surgery. Based on documented follow-up modes, patients were categorized into three groups: telephone, WeChat, and the “Doctor Haixin” mini-program. Key clinical indicators, including treatment adherence, quality of life, adverse events, and self-management levels, were extracted from medical records and patient-reported follow-up data collected over a 12-week period.

**Results:**

Patients in the mini-program group were found to have higher recorded adherence rates and better self-reported outcomes in quality of life and self-management compared with the telephone and WeChat groups. All groups showed improved quality of life over time, while adverse event rates remained comparable across groups.

**Conclusion:**

Among patients retrospectively assessed, those managed via the mini-program follow-up exhibited more favorable patterns in adherence and patient-reported outcomes. These findings suggest that digital platforms may be associated with enhanced care quality in the context of breast cancer follow-up.

## 1 Introduction

Breast cancer is one of the most common female malignant tumors in the world, and its incidence and fatality rate are increasing year by year, causing a huge impact on public health. With the improvement of medical level, early diagnosis and treatment of breast cancer are gradually improved (1). Adjuvant therapy for breast cancer, including endocrine therapy, chemotherapy, radiotherapy, and targeted therapy, is administered after surgery to minimize the risk of tumor recurrence and extend patient survival. This approach effectively eliminates residual cancer cells, helps maintain long-term disease stability, and significantly enhances survival rates (2–4). In the adjuvant treatment of breast cancer, treatment compliance not only determines the individual efficacy, but also profoundly affects the long-term prognosis and quality of life of patients (5). Treatment compliance refers to the extent to which patients follow the recommended treatment plan, including taking medication on time, regular checkups, and receiving recommended treatment. However, some breast cancer patients are affected by such factors as lack of health knowledge, poor economic status, side effects of drugs, poor mental state, and long duration of adjuvant treatment (6). At present, compliance with adjuvant therapy for breast cancer is a common problem in the world. About 20%-50% of patients fail to fully comply with the endocrine therapy plan, and domestic studies also reflect similar problems (7, 8). Despite advancements in medical resources, poor adherence remains a significant challenge in breast cancer treatment. Implementing comprehensive management interventions, establishing structured follow-up programs, and developing patient-centered support systems are essential for enhancing treatment compliance and safety. These measures can ultimately help breast cancer patients achieve longer survival and an improved quality of life.

In recent years, there are many follow-up programs to improve compliance with adjuvant therapy for breast cancer, including telephone follow-up and Wechat follow-up, which have shown potential advantages, but telephone follow-up is labor-intensive and cannot provide long-term and traceable records. Wechat follow-up cannot fully convey accuracy (9, 10). With the development of artificial intelligence and its gradual application to the medical field, the Chinese Society of Clinical Oncology (CSCO) has developed a small program for the treatment and management of cancer patients “Doctor Haixin”, which connects doctors and patients, so that more patients can enjoy standard and refined diagnosis and treatment services. However, at present, there is no systematic report on the management and application effect of this small program in the adjuvant therapy of breast cancer patients, and whether it can improve the treatment compliance and safety of patients remains to be further verified. This study retrospectively analyzed the associations between different documented follow-up management methods—telephone, WeChat, and the Doctor Haixin mini-program—and treatment compliance and safety among breast cancer patients undergoing adjuvant therapy.

## 2 Data and methods

### 2.1 General information

This retrospective study included 180 female patients with newly diagnosed breast cancer who were admitted to our hospital between January 2022 and December 2024. Eligible patients met the following criteria based on medical record review:

1. Histopathological confirmation of breast cancer following radical surgery (excluding certain invasive subtypes);
2. Clinical stage I–III;
3. No evidence of distant metastasis on imaging;
4. Estimated life expectancy over six months at diagnosis;
5. Documented ability to use a smartphone.

Patients were excluded from analysis if their records indicated:

1. Male, bilateral, inflammatory, or pregnancy/lactation-associated breast cancer;
2. A history of other malignancies;
3. Receipt of prior chemotherapy or radiotherapy;
4. Incomplete adjuvant therapy;
5. Diagnosed psychiatric disorders or cognitive impairment;
6. Disorders of consciousness or severe systemic comorbidities;
7. Incomplete clinical or follow-up data.

Additionally, cases were excluded from final analysis if follow-up records showed:

1. Voluntary discontinuation of treatment;
2. Loss to follow-up;
3. Severe adverse events preventing completion of therapy;
4. Termination of adjuvant therapy for any reason;
5. Death prior to follow-up endpoint.

### 2.2 Grouping and method

The 180 included patients were retrospectively categorized into three groups based on the documented follow-up method recorded in their clinical records: telephone group (n = 60), WeChat group (n = 60), and mini-program group (n = 60). Follow-up methods consisted of telephone-based contact, WeChat-based communication, and use of the “Doctor Haixin” digital mini-program, respectively. Patients were retrospectively categorized into the telephone, WeChat, or mini-program groups based on the follow-up method documented in their clinical records. The follow-up method was determined by patient preference and clinician recommendation at the time of initial treatment planning. As this was not a randomized process, potential selection bias cannot be excluded.

Baseline clinical information was extracted for all patients, including age, body mass index (BMI), clinical stage, tumor laterality, surgical procedure, estrogen receptor (ER) and progesterone receptor (PR) status, menstrual history, pathological type, human epidermal growth factor receptor (HER) and HER2 status, as well as adjuvant treatment details.

This retrospective study was approved by the Ethics Committee of our hospital (Approval No. HUH202504121). Written informed consents from all patients were obtained in any experimental work with humans.

### 2.3 Method

According to clinical documentation, patients routinely received health education upon hospital admission, which typically included an overview of treatment goals, therapeutic options, potential side effects and corresponding management strategies, daily lifestyle recommendations, exercise guidance, and psychological support.

#### 2.3.1 Treatment plan

All patients underwent radical mastectomy performed by the same surgical team at our hospital. Postoperative adjuvant therapy regimens were determined according to the CSCO guidelines for breast cancer (11), and were individualized based on receptor status:

- HER2-negative regimen: Doxorubicin (60 mg/m²) plus cyclophosphamide (600 mg/m²) administered intravenously every 3 weeks for 4 cycles (12 weeks), followed by paclitaxel (80 mg/m²) weekly for 12 cycles.
- HER2-positive regimen: Doxorubicin and cyclophosphamide as above, combined with trastuzumab (8 mg/kg loading dose, 6 mg/kg maintenance every 3 weeks) for a total of 1 year.
- ER and/or PR-positive patients: Received tamoxifen (20 mg/day) orally for 5 years.

Patients were retrospectively grouped according to the documented follow-up method used during adjuvant therapy, which included telephone follow-up, WeChat-based follow-up, or use of the “Haixin Doctor” mini program. The specific contents of each follow-up mode were obtained from archived follow-up logs and nursing records.

#### 2.3.2 Telephone follow-up group

According to clinical follow-up documentation, patients in this group received biweekly telephone follow-up calls. Records indicate that:

1. During initial contact, staff verified basic patient information (e.g., diagnosis, medications, lifestyle) and provided guidance on medication use, symptom monitoring, and side effect management.
2. Routine follow-up included evaluation of treatment adherence and physical condition. Adverse events and patient concerns were noted, and patients were referred to physicians as needed.
3. For severe symptoms or emotional distress, emergency referrals were documented and acted upon according to hospital policy.

#### 2.3.3 WeChat follow-up group

Follow-up records for this group reflected WeChat-based communication as the primary mode. According to the logs:

1. Patients were added to a WeChat contact list and provided with electronic health education materials.
2. Weekly interactions via text, voice, or video included medication compliance questionnaires, symptom tracking forms, and personalized feedback.
3. Health tips and reminders were sent regularly regarding diet, exercise, and follow-up schedules.

#### 2.3.4 Mini program follow-up group

Patients in this group were recorded as having used the “Haixin Doctor” mini program. Clinical notes and app logs documented:

1. Initial registration assistance and training on how to use the app features (e.g., medication reminders, symptom reporting, educational content).
2. Patients submitted weekly questionnaires and logged daily health indicators such as weight, blood pressure, and symptom reports.
3. Questions submitted through the app were answered by medical staff within 24 hours.
4. The system triggered alerts to healthcare providers if serious symptoms or side effects were reported.

#### 2.3.5 System monitoring and data feedback

The mini program automatically generated patient health summaries based on input data. According to institutional policy, these reports were reviewed by clinicians to monitor trends in compliance and patient-reported outcomes. Adjustments to treatment or health management were recorded when clinically indicated.

#### 2.3.6 Follow-up duration and contact records

All patients were followed for a standard 12-week period based on their adjuvant therapy regimen. Contact attempt records showed that if patients could not be reached through the primary method (e.g., WeChat or the mini program), follow-up was attempted via telephone. A maximum of three call attempts were recorded, spaced at least one day apart. If unsuccessful, further attempts were made via family members or local community health services.

### 2.4 Treatment compliance

Treatment compliance was retrospectively evaluated based on documented clinical records and follow-up logs over a 12-week treatment period. The criteria for classification were adapted from previously published literature (12):

- Complete compliance: Medical records indicated that the patient completed the prescribed chemotherapy regimen according to medical recommendations, including the appropriate dosage and schedule. The patient exhibited proactive behavior toward treatment, was reachable during all follow-up attempts, and there were no recorded delays, interruptions, or cancellations.
- Partial compliance: Patients completed treatment only with external supervision or reminders (e.g., from physicians or family members). Follow-up documentation showed more than five successful contacts, with ≥2 delays or ≥2 instances of treatment interruption/cancellation recorded.
- Non-compliance: Patients failed to complete the prescribed chemotherapy regimen as documented. Records reflected fewer than three successful follow-up contacts, more than three delays, and ≥3 interruptions or cancellations during the treatment period.

The overall compliance rate was calculated using the formula: (number of patients with complete compliance + partial compliance)/total number of patients × 100%.

### 2.5 Side reaction comparison

Adverse events recorded during the 12-week treatment period were retrospectively analyzed based on patients’ medical records and laboratory results. The classification of toxic reactions followed the World Health Organization (WHO) standard criteria for evaluating adverse reactions to anticancer drugs (13). The documented side effects included:

- Gastrointestinal reactions: Such as nausea, vomiting, anorexia, diarrhea, or constipation, as recorded in clinical progress notes or nursing reports.
- Myelosuppression: Defined by documented reductions in peripheral blood counts, including white blood cells, red blood cells, and platelets, based on laboratory test reports.
- Allergic reactions: Manifested as rashes, pruritus, or dyspnea, as noted in medical or nursing records.
- Cardiotoxicity: Identified through decreased ejection fraction measurements reported in echocardiography records.

### 2.6 Quality of life

Patients’ quality of life at baseline and after 12 weeks of follow-up was retrospectively assessed using the Functional Assessment of Cancer Therapy-Breast (FACT-B) scale (14), as recorded in clinical follow-up documentation. The FACT-B consists of the general Functional Assessment of Cancer Therapy (FACT) and the Breast Cancer Subscale (BCS), comprising five dimensions: physical well-being (7 items), social/family well-being (7 items), emotional well-being (6 items), functional well-being (7 items), and breast cancer–specific concerns (9 items), totaling 36 items. Each item is scored on a 5-point Likert scale (0–4), with higher total scores indicating better quality of life.

### 2.7 Self-management ability

Patients’ self-management ability was retrospectively assessed using the Cancer Patient Self-Management Assessment Scale (15), based on follow-up records collected at baseline and after 12 weeks. The scale includes six dimensions: self-efficacy (10 items), daily life management (11 items), access to information (3 items), symptom management (7 items), communication with healthcare providers (4 items), and psychological adjustment (9 items). Higher scores reflect stronger self-management capabilities.

### 2.8 Study flow chart

**Figure 1:**
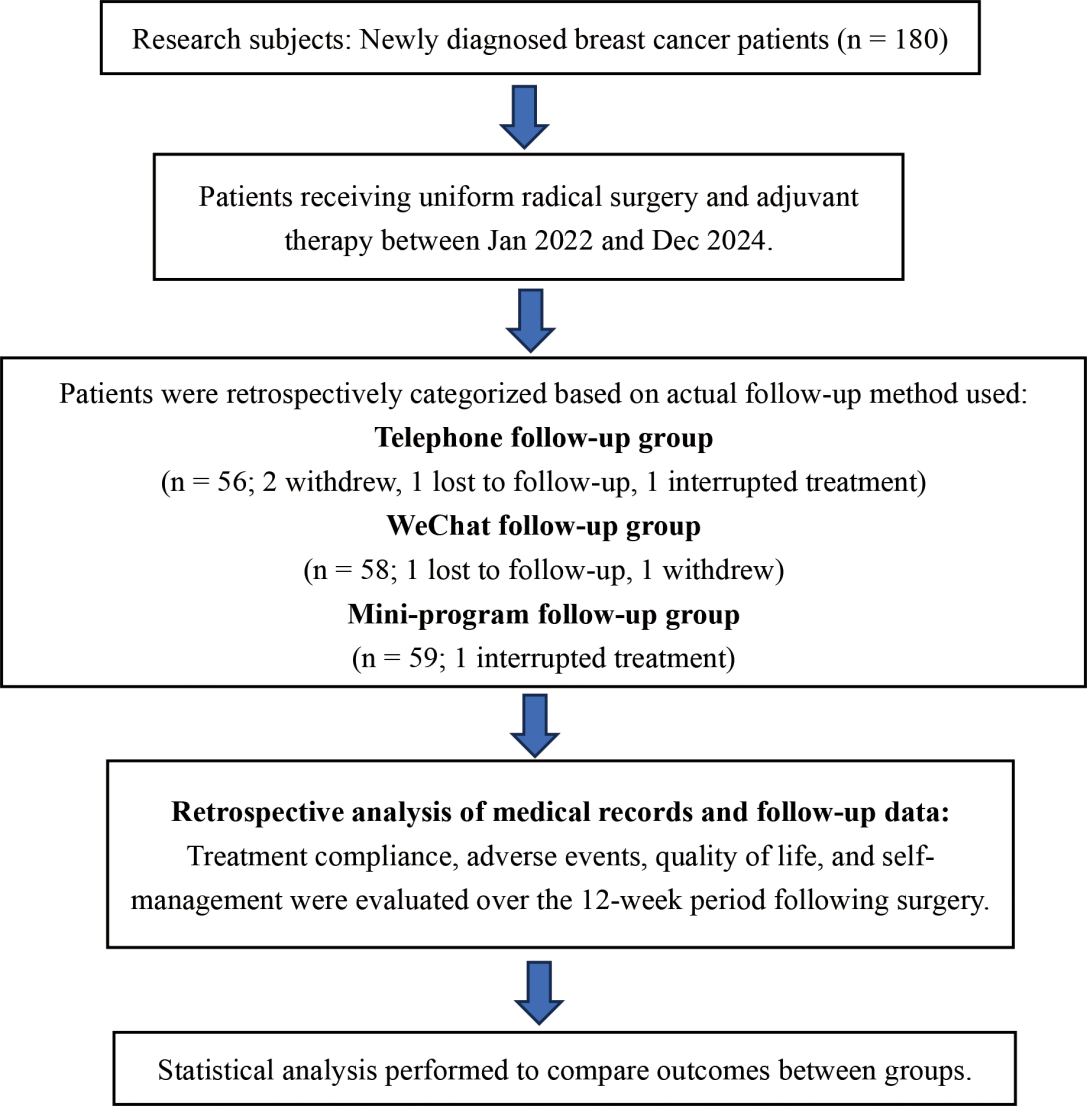
Research flow chart.

**Figure 1** shows the flow chart of this research.

### 2.9 Statistical analysis

SPSS26.0 statistical software was used for data analysis, images were processed by Prism, and the measurement data conforming to normal distribution were expressed by mean ± standard deviation (). The comparison at different time points was performed by inter-group, time and interactive repeated measurement ANOVA, and the pair comparison was performed by LSD-t test. The statistical data were represented by [n (%)], χ^2^ test or Fisher exact test were used for comparison between groups, and Bonferroni correction was used for further pairwise comparison. *P* < 0.05 was considered statistically significant (bilateral test).

## 3 Results

### 3.1 Comparison of clinical data

In this study, the age range was 40 to 55 years old, with an average age of (43.92 ± 3.10) years. In the telephone group, 2 cases withdrew from the study voluntarily, 1 case was lost to follow-up, and 1 case was interrupted by adjuvant therapy. In the Wechat group, 1 case was lost to follow-up and 1 case withdrew from the study. One case of interruption of adjuvant therapy in the small program group. Through the observation of general data, no differences were observed among the three groups in age, BMI, clinical stage, affected side, surgical method, ER expression, PR expression, menstrual status, pathological type, HER expression, HER2 expression, and adjuvant treatment regimen (P>0.05), as shown in **Table 1**.

**Table 1:** Comparison of clinical data.

Characteristics	Telephone group (*n* = 56)	Wechat group (*n* = 58)	Small program group (*n* = 59)	Statistical value	*P*
Age (years)	43.41 ± 3.10	44.39 ± 3.08	43.97 ± 3.13	1.427^①^	0.243
BMI (kg/m²)	21.20 ± 1.25	21.17 ± 1.23	21.19 ± 1.24	0.009^①^	0.991
Clinical stage				0.721^②^	0.697
Phase I and II	31 (57.41)	29 (50.00)	34 (57.63)		
Phase III	25 (46.30)	29 (50.00)	25 (42.37)		
Affected side				2.884^②^	0.236
Left side	34 (62.96)	26 (44.83)	31 (52.54)		
Right side	22 (40.74)	32 (55.17)	28 (47.46)		
Mode of operation				4.905^②^	0.086
Breast preservation	21 (38.89)	17 (29.31)	29 (49.15)		
Non-breast-conserving surgery	35 (64.81)	41 (70.69)	30 (69.49)		
ER expression				3.918^②^	0.141
Masculine	21 (38.89)	12 (20.69)	18 (30.51)		
Feminine character	45 (83.33)	46 (79.31)	41 (69.49)		
PR expression				4.202^②^	0.122
masculine	17 (31.48)	11 (18.97)	9 (15.25)		
Feminine character	39 (72.22)	47 (81.03)	50 (84.75)		
Menstrual state				1.923^②^	0.382
Menopause	6 (11.11)	11 (18.97)	7 (11.86)		
Premenopause	50 (92.59)	47 (81.03)	52 (88.14)		
Pathological type				0.282^③^	0.991
Invasive ductal carcinoma	35 (64.81)	39 (67.24)	39 (66.10)		
Invasive lobular carcinoma	11 (20.37)	10 (17.24)	11 (18.64)		
Invasive carcinoma	10 (18.52)	9 (15.52)	10 (16.95)		
HER expression				3.738	0.154
masculine	17 (31.48)	14 (24.14)	9 (15.25)		
Feminine character	39 (85.19)	44 (75.86)	50 (84.75)		
HER2 expression				1.363	0.506
masculine	10 (18.52)	15 (25.86)	11 (18.64)		
Feminine character	46 (85.19)	43 (74.14)	48 (81.36)		
Adjuvant treatment program				9.708^③^	0.050
chemotherapy	18 (33.33)	32 (55.17)	32 (54.24)		
Targeted therapy	17 (31.48)	14 (24.14)	9 (15.25)		
Endocrine therapy	21 (38.89)	12 (20.69)	18 (30.51)		

^①^ is F test, ^②^ is χ^2^ test, ^③^ Fisher test.

### 3.2 Comparison of adjuvant treatment compliance among all groups

**Figure 2:**
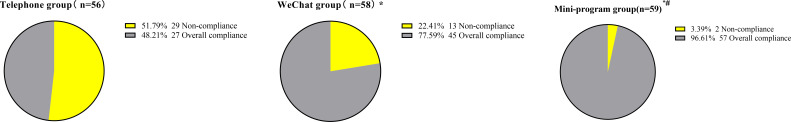
Comparison of adjuvant treatment compliance among the groups (compared with the phone group ^*^P < 0.05, compared with the Wechat group ^#^*P* < 0.05).

The overall compliance of the three groups was significantly different (P<0.05). Further pair-to-pair comparison by Bonferroni correction showed that the overall compliance of the telephone group was lower than that of the Wechat group and the mini program group (P<0.05). The overall compliance of the Wechat group was lower than that of the small program group (P<0.05), as shown in **Table 2** and **Figure 2**.

**Table 2:** Comparison of adjuvant treatment compliance among all groups.

Materials	Complete compliance	Partial compliance	Noncompliance	Overall compliance
Telephone group (*n* = 56)	12 (21.49)	15 (26.79)	29 (51.79)	27 (48.21)
Wechat group (*n* = 58)	21 (36.21)	24 (41.37)	13 (22.41)	45 (77.59)^*^
Small program group (*n* = 59)	31 (52.54)	26 (44.07)	2 (3.39)	57 (96.61)^*#^
*χ^2^*				35.902
*P*				<0.001

Compared with the phone group ^*^P < 0.05, compared with the Wechat group ^#^*P* < 0.05.

### 3.3 The occurrence of side reactions in each group was compared

**Figure 3:**
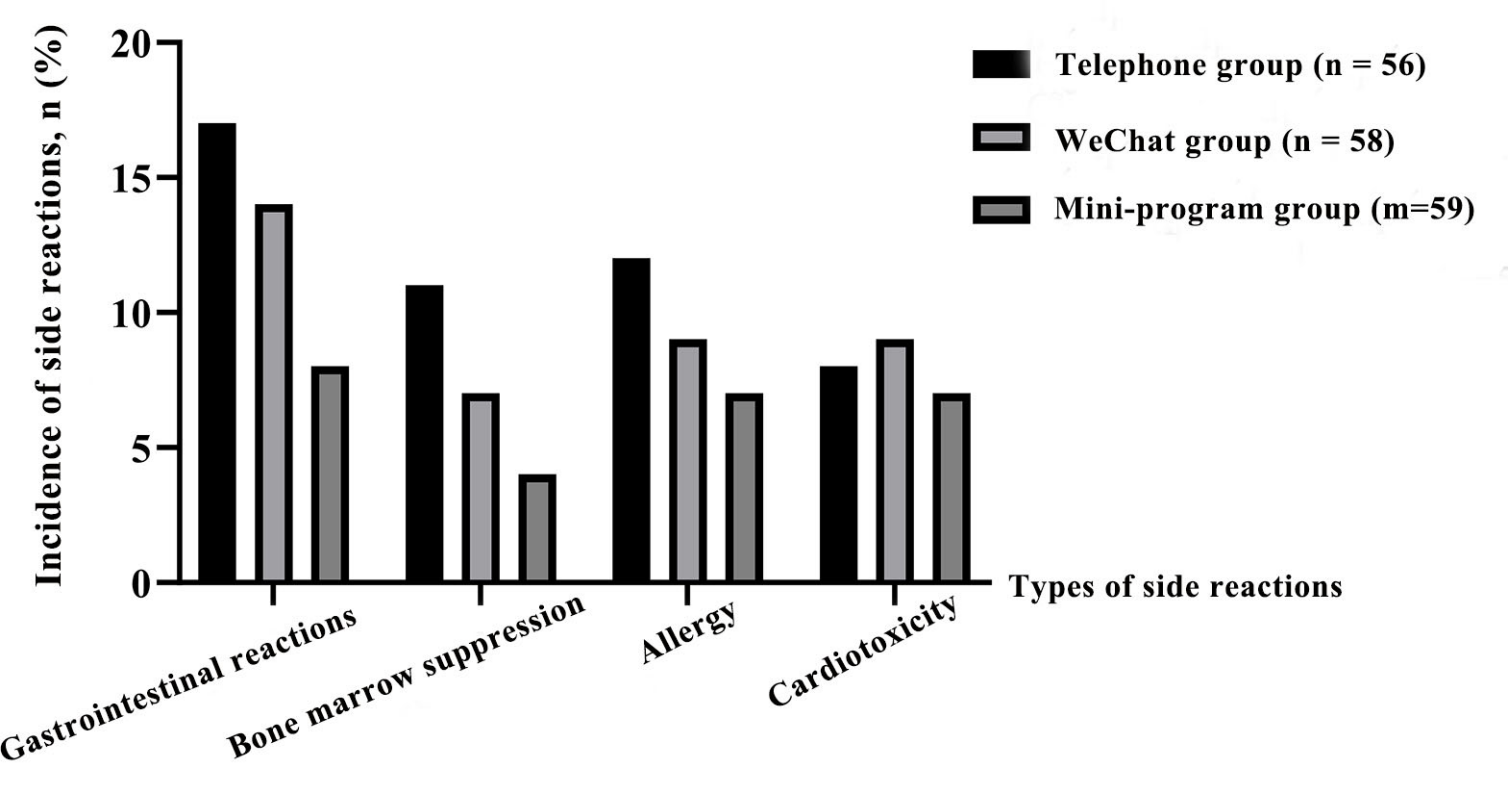
Comparison of the occurrence of side reactions in each group.

There were no significant differences in gastrointestinal reaction, bone marrow suppression, allergy and cardiotoxicity among the three groups (P > 0.05). Further pound-to-pair comparison by Bonferroni correction showed that there were no significant differences in gastrointestinal reactions, bone marrow suppression, allergy and cardiotoxicity between the phone group and the Wechat group, the phone group, and the mini program group, and the Wechat mini program group (P > 0.05), as shown in **Table 3** and **Figure 3**.

**Table 3:** Comparison of the occurrence of side reactions in each group.

Groups	Gastrointestinal reaction	Myelosuppression	Allergy	Cardiotoxicity
Telephone group (*n* = 56)	17 (30.36)	11 (19.64)	12 (21.43)	8 (14.29)
Wechat group (*n* = 58)	14 (24.14)	7 (12.07)	9 (15.52)	9 (15.52)
Small program group (*n* = 59)	8 (13.56)	4 (6.78)	7 (11.86)	7 (11.86)
*χ^2^*	4.769	4.316	2.173	0.338
*P*	0.092	0.116	0.337	0.844

### 3.4 Comparison of quality of life in each group

**Figure 4:**
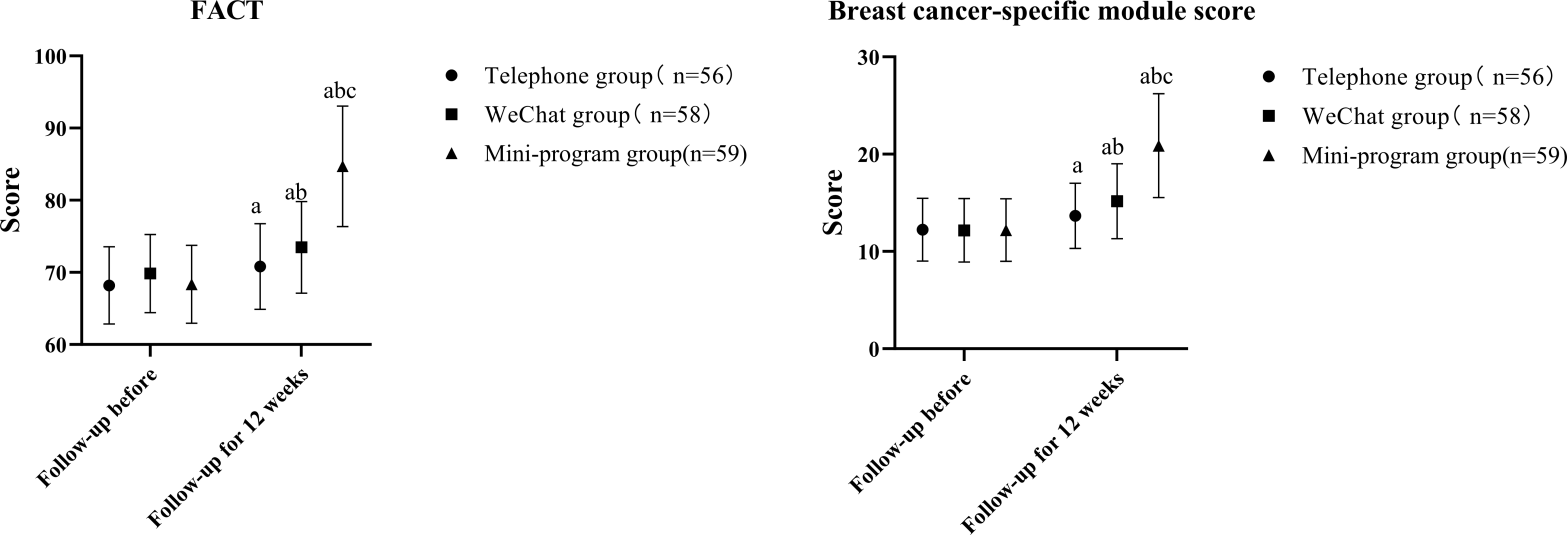
Comparison of various quality of life (compared with pre-follow-up, ^a^*P* < 0.05, compared with telephone group, ^b^*P* < 0.05, compared with wechat group, ^c^*P* < 0.05).

Before follow-up, there were no differences in FACT score and breast cancer specific module score among all groups in one-way ANOVA (P>0.05), but there were significant differences in FACT score and breast cancer specific module score among all groups at 12 weeks follow-up (P<0.05). After 12 weeks of follow-up, compared with before follow-up, the FACT scores and breast cancer specific module score of all groups were increased, with certain differences (P<0.05). Compared with the phone group, the FACT score, and breast cancer specific module score were higher in the Wechat group and the mini program group (P<0.05), while the FACT scores and breast cancer specific module score in the mini program group were higher than those in the Wechat group, with significant differences (P<0.05), as shown in **Table 4** and **Figure 4**.

**Table 4:** Comparison of various quality of life.

Groups	FACT rating		Breast cancer specific module score	
	Pre-follow-up	Follow-up for 12 weeks	Pre-follow-up	Follow-up for 12 weeks
Telephone group (*n* = 56)	68.19 ± 5.35	70.82 ± 5.93^a^	12.24 ± 3.23	13.67 ± 3.35^a^
Wechat group (*n* = 58)	69.84 ± 5.42	73.47 ± 6.34^ab^	12.17 ± 3.26	15.16 ± 3.85^ab^
Small program group (*n* = 59)	68.35 ± 5.39	84.71 ± 8.35^abc^	12.20 ± 3.21	20.87 ± 5.34^abc^
*F*	1.646	64.918	0.007	45.736
*P*	0.196	<0.001	0.993	<0.001

Compared with before follow-up, ^a^*P* < 0.05, ^b^*P* < 0.05 compared with phone group, ^c^*P* < 0.05 compared with Wechat group.

### 3.5 Comparison of self-management ability in each group

Before follow-up, there were no differences in scores of self-management ability among all groups in univariate analysis of variance (P>0.05). After 12 weeks of follow-up, there were significant differences in self-efficacy, daily life, information, communication with medical staff and psychological comparison among all groups (P<0.05). Compared with before follow-up, there were significant differences in self-efficacy and daily life in Wechat group and mini program group after 12 weeks of follow-up (P<0.05), while there were no differences in self-efficacy and daily life in phone group after 12 weeks of follow-up (P>0.05). After 12 weeks of follow-up, the information, communication with medical staff and psychological scores of the three groups were increased (P<0.05). There was no difference between the phone group and the Wechat group in self-efficacy, daily life and information (P>0.05), while the mini program group had higher self-efficacy, daily life, information, communication with medical staff and psychological scores than the phone group and mini program group (P<0.05), while there was no significant difference in symptoms among the three groups (P>0.05), as shown in **Table 5**.

**Table 5:** Comparison of self-management ability among groups.

Dimensions of self-management ability	Time	Telephone group (*n* = 56)	Wechat group (*n* = 58)	Small program group (*n* = 59)	*F*	*P*
Self-efficacy	pre-follow-up	24.19 ± 2.34	24.20 ± 2.37	23.97 ± 2.30	0.181	0.835
	Follow-up for 12 weeks	25.11 ± 2.56	25.20 ± 2.59^a^	30.45 ± 4.61^abc^	46.899	<0.001
Daily life	pre-follow-up	31.10 ± 4.92	30.82 ± 4.89	31.12 ± 4.95	0.067	0.945
	Follow-up for 12 weeks	32.19 ± 5.02	33.21 ± 4.95^a^	37.18 ± 6.78^abc^	12.586	<0.001
Message	pre-follow-up	3.11 ± 0.72	3.23 ± 0.81	3.17 ± 0.75	0.354	0.702
	Follow-up for 12 weeks	3.85 ± 0.89^a^	4.14 ± 0.95^a^	7.38 ± 1.28^abc^	200.029	<0.001
Symptom	pre-follow-up	9.91 ± 0.97	9.97 ± 0.99	9.92 ± 1.04	0.174	0.841
	Follow-up for 12 weeks	10.24 ± 1.15	10.31 ± 1.17	10.17 ± 1.20	0.804	0.584
Communicate with medical staff	pre-follow-up	2.70 ± 0.24	2.9 ± 0.25	2.74 ± 0.27	0.635	0.531
	Follow-up for 12 weeks	6.15 ± 0.39^a^	10.84 ± 2.11^ab^	15.28 ± 3.21^abc^	236.767	<0.001
mind	pre-follow-up	21.81 ± 3.56	20.71 ± 3.35	22.05 ± 3.75	2.343	0.099
	Follow-up for 12 weeks	23.25 ± 3.61^a^	27.82 ± 4.37^ab^	30.71 ± 5.14^abc^	41.261	<0.001

Compared with before follow-up, ^a^*P* < 0.05, ^b^*P* < 0.05 compared with phone group, ^c^*P* < 0.05 compared with wechat group.

## 4 Discussion

In recent years, with the improvement of breast cancer screening work and diagnosis and treatment technology, the survival period of breast cancer groups has been extended with the assistance of various programs such as surgery, chemoradiotherapy, targeted therapy and endocrine therapy. However, patients are faced with related adjuvant therapy and mental health changes after surgery, and the continuous management of these patients has become a research focus (16–18). In addition to regular follow-up and medical interventions by physicians, continuity of care extends beyond the hospital setting. Integrating medical and nursing care throughout the entire treatment process helps patients navigate postoperative challenges, manage psychological distress, and ultimately enhance their quality of life and long-term prognosis (19). Therefore, breast cancer patients need long-term out-of-hospital follow-up management. Effective follow-up not only provides rehabilitation guidance but also enables monitoring of tumor progression, treatment-related side effects, and psychological support. However, traditional follow-up methods—such as telephone calls, text messages, WeChat follow-ups, and public account notifications—have limitations, making it challenging to achieve comprehensive management for patients undergoing adjuvant therapy for breast cancer (20, 21). Therefore, it is imperative to find a new and effective follow-up management program.

“Doctor Haixin” mini program is a medical and health program, usually used to provide online consultation, health consultation, appointment registration, health management and other services, through digital means to improve patients’ treatment compliance and health management effects. Studies have shown that the use of digital tools (such as mini programs) can significantly improve the treatment compliance of breast cancer patients, which is the key to improving patient prognosis and quality of life (22, 23). The results revealed significant differences in overall compliance among the three patient groups, with adherence levels ranked from lowest to highest as follows: telephone group < WeChat group < mini program group. Compliance was lowest in the telephone follow-up group, likely due to the inherent limitations of phone-based communication. Telephone follow-ups are constrained by time, lack intuitive information delivery, and do not allow patients to revisit the follow-up content easily. Additionally, the interaction frequency between patients and follow-up personnel is relatively low (24, 25). WeChat follow-up demonstrated higher compliance than telephone follow-up, benefiting from its immediacy and multimedia functionality. The ability to communicate via text, voice messages, and images enables real-time interaction, while educational materials can be easily saved and revisited, enhancing patient engagement and adherence (26). “Doctor Haixin” mini program has the highest follow-up compliance, due to its comprehensiveness and data visualization. The mini program not only provides health education and follow-up questionnaires, but also supports patients to record health data, medication reminders, online consultation and other functions, enhancing patients’ sense of participation and self-management ability.

In terms of safety, there were no significant differences in gastrointestinal reactions, bone marrow suppression, allergies, cardiotoxicity, and other adverse reactions among the three groups. Adverse reactions are mainly determined by the breast cancer treatment program itself, and the direct correlation with the follow-up method is weak. Although the follow-up method has no significant impact on the incidence of adverse reactions, timely intervention (such as medication guidance, side effect management, etc.), “Doctor Haixin” mini program and Wechat follow-up may better help patients alleviate the symptoms of adverse reactions. Before follow-up, there were no significant differences in FACT scores and breast cancer specific module scores among all groups. After 12 weeks of follow-up, the scores of all groups were significantly increased, and the small program group was > wechat group > telephone group. The improvement of telephone follow-up was small, which may be due to the low frequency of follow-up and the limited health education and personalized guidance received by patients (27, 28). Through the diversified forms of communication on Wechat, patients can get more health education and personalized advice, which improves the quality of life. The “Doctor Haixin” mini program group has the largest improvement, and the mini program has comprehensive functions and high interaction frequency. Through daily health data recording and online consultation at any time, patients can better understand and manage their health conditions, and enhance treatment confidence and the advantages of the life quality program. “Doctor Haixin” mini program integrates health data record, medication reminder, follow-up questionnaire, health education push and online consultation and other functions, so that patients’ health management is more systematic. By automatically generating health data reports, patients and follow-up staff can intuitively understand changes in health status and adjust management plans (29). Small programs support patients to submit questions at any time, and follow-up staff can give feedback within 24 hours, enhancing patients’ sense of security and trust (30).

Recent studies increasingly support the role of digital health platforms in improving treatment adherence and patient self-management among individuals with breast cancer and other chronic conditions. For example, a 2025 randomized clinical trial demonstrated that a self-management mobile application significantly improved quality of life among women with breast cancer-related lymphedema, underscoring the potential for structured digital support to deliver tangible health benefits (31). Similarly, a non-randomized intervention trial of the CAMA app, published in 2025, showed promising improvements in self-efficacy, psychological well-being, and anxiety reduction in breast cancer survivors, echoing the type of benefits observed in our study (32). Moreover, a 2025 quasi-experimental mobile health coaching program in South Korea demonstrated significant enhancements in self-management, symptom burden, emotional well-being, and overall quality of life over 12 weeks, further aligning with our findings (33). By contrast, a 2025 umbrella review of digital health interventions in breast cancer found that while interventions generally show benefits, outcomes vary considerably depending on intervention design, delivery modality, and duration, suggesting that comprehensive features as offered by the “Doctor Haixin” mini-program may be instrumental in sustaining engagement and adherence (34). Together, these findings suggest that digital follow-up tools represent a promising approach to enhancing patient-centered care, although their design and implementation may influence outcomes.

The follow-up period of this study was 12 weeks, and the long-term follow-up effect could not be observed to evaluate the continuing role of mini programs in long-term health management. The treatment regimen and condition of breast cancer patients vary greatly, and the uniform follow-up model of small programs may not fully meet the individual needs. In the future, the follow-up time will be extended, and the adjuvant treatment regimen will be unified to further explore the results.

## 5 Limitations

This study has several limitations. First, its retrospective design restricts the ability to establish causal relationships between follow-up methods and patient outcomes. The findings demonstrate associations rather than causation and should be interpreted with caution. Future studies should adopt prospective or randomized controlled trial designs to better evaluate causal relationships. Second, the follow-up period was limited to 12 weeks, which does not allow assessment of long-term adherence or quality-of-life outcomes. Future research should incorporate extended follow-up durations to evaluate the sustainability of digital interventions and their long-term effects on adherence, quality of life, and patient-reported outcomes. Third, as this was a single-center study, the generalizability of the findings may be limited. Future studies should consider multicenter designs involving diverse populations to improve external validity. Additionally, because patients were not randomly assigned to follow-up methods, the possibility of selection bias exists. Factors such as patient preference, clinician discretion, age, digital literacy, and socioeconomic status could have influenced both the choice of follow-up method and the outcomes observed. Future investigations should control for these potential confounders using stratification, matching techniques, or multivariable adjustment models. Furthermore, patients who were unable to use smartphones were excluded from this study, which limits the applicability of the findings to digitally underserved populations. Future research should explore hybrid or inclusive follow-up approaches that accommodate patients with varying levels of digital literacy and access to technology. Finally, the uniform follow-up structure provided by the “Doctor Haixin” mini-program may not fully meet the individualized needs of all patients. Future studies should evaluate the impact of more personalized, adaptive digital health interventions to optimize patient engagement and outcomes.

## 6 Conclusion

The “Doctor Haixin” mini-program was associated with favorable outcomes in the follow-up management of breast cancer patients, particularly in terms of treatment compliance and patient-reported quality of life. Compared with traditional telephone and WeChat follow-up, the mini-program provided more efficient interaction and comprehensive management between patients and healthcare providers through digital means. However, given the retrospective design of this study, causal relationships cannot be established, and the findings should be interpreted cautiously. Future prospective, multicenter studies with longer follow-up periods are needed to validate these associations and further explore the effectiveness of digital health platforms. In the future, mini-program functions can also be optimized and potentially integrated with artificial intelligence technology to provide patients with more personalized and accurate health management services.

## Data Availability

The raw data supporting the conclusions of this article will be made available by the authors, without undue reservation.
