# Correction: Evaluation of real-world follow-up methods and their association with adherence and safety in adjuvant breast cancer therapy: a retrospective study

**DOI:** 10.3389/fonc.2025.1774191

**Published:** 2026-01-13

**Authors:** Na Li, Qian Zhang, Xiaomei Yang, Xiaoli Li, Xixi Tian, Wei Li, Lili Ren, Hua Yang

**Affiliations:** 1 Department of Medical Oncology, Affiliated Hospital of Hebei University, Baoding, Hebei, China; 2 Hebei Key Laboratory of Cancer Radiotherapy and Chemotherapy, Baoding, Hebei, China; 3 Teaching Office, Affiliated Hospital of Hebei University, Baoding, Hebei, China

**Keywords:** breast cancer, treatment compliance, digital follow-up, self-management, patient-reported outcomes

In the **Funding** Section, an incorrect grant number was provided for the “Hebei Provincial Health Commission Medical Science Research Project”. The correct grant number is “NO. 20231514”.

The original version of this article has been updated.

